# Elevated circulating activin A is associated with disease severity in advanced steatotic liver disease (SLD)

**DOI:** 10.1186/s12876-026-05550-6

**Published:** 2026-11-10

**Authors:** Karin Hedin, Patrik Nasr, Fredrik Iredahl, Mikael Forsgren, Karin Rådholm, Nils Dahlström, Henrik Stjernman, Daniel Sjögren, Martin Rejler, Olof Dahlqvist Leinhard, Peter Lundberg, Stergios Kechagias, Cecilia Jönsson, Mattias Ekstedt

**Affiliations:** 1 Department of Health, Medicine and Caring Sciences (HMV), Linköping University, Linköping, Sweden; 2 Wallenberg Center for Molecular Medicine, Linköping University, Linköping, Sweden; 3 Center for Medical Image Science and Visualization (CMIV), Linköping University, Linköping, Sweden; 4 Primary Health Care Center, Department of Health, Medicine and Caring Sciences (HMV), Faculty of Medicine and Health Sciences, Linköping University, Linköping, Sweden; 5 AMRA Medical AB, Linköping, Sweden; 6 Primary Health Care Center Kärna, Department of Health, Medicine and Caring Sciences (HMV), Linköping University, Linköping, Sweden; 7 The George Institute for Global Health, University of New South Wales, Sydney, Australia; 8 Department of Radiology, Department of Health, Medicine and Caring Sciences (HMV), Linköping University, Linköping, Sweden; 9 Department of Internal Medicine, Ryhov County Hospital Jönköping, Jönköping, Sweden; 10 Department of Medicine, Höglandssjukhuset Eksjö, Region Jönköping County Council, Jönköping, Sweden; 11 Jönköping Academy for Improvement of Health and Welfare, Jönköping University, Jönköping, Sweden; 12 Futurum Academy for Healthcare, Region Jönköping County, Jönköping, Sweden; 13 Department of Radiation Physics, Department of Health, Medicine and Caring Sciences (HMV), Linköping University, Linköping, Sweden; 14 Clinical Department of Gastroenterology and Hepatology in Linköping, Region Östergötland, Linköping, Sweden; 15 SciLifeLab, Linköping University, Linköping, Sweden

**Keywords:** Activin A, Follistatin, Metabolic dysfunction-associated steatotic liver disease (MASLD), Steatotic liver disease (SLD), Fibrosis, Cirrhosis

## Abstract

**Background:**

Steatotic liver disease (SLD) is a major contributor to the global burden of chronic liver disease and is linked to liver fibrosis. Emerging evidence implicates activin A in this process, but its role remains unclear due to conflicting findings. This cross-sectional study examines circulating levels of activin A and its endogenous inhibitor follistatin in individuals with metabolic dysfunction and evaluates their associations with clinical indicators of steatosis and fibrosis.

**Methods:**

Plasma activin A and follistatin were measured with ELISA in 309 participants with type 2 diabetes (T2D) with or without SLD and in 97 participants with SLD-related cirrhosis. Hepatic fat was assessed using ^1^H-MRS-PDFF or MRI-PDFF, or if unavailable, controlled attenuation parameter (CAP). Liver stiffness was measured with vibration-controlled transient elastography (VCTE).

**Results:**

In the diabetes cohort, participants with steatosis and fibrosis had higher activin A concentrations than participants with neither steatosis nor fibrosis (0.400 ng/mL vs. 0.285 ng/mL, *P* = 0.036). In the cirrhosis cohort, activin A correlated strongly with liver stiffness (ρ = 0.696, *P* < 0.001). Participants with VCTE suggestive of compensated advanced chronic liver disease (cACLD) and clinically significant portal hypertension (CSPH) exhibited significantly higher activin A concentrations than participants without (0.546 vs. 0.307 ng/mL, *P* < 0.001 and 0.624 vs. 0.396 ng/mL, *P* < 0.001, respectively). In this study, neither follistatin nor the activin A-to-follistatin ratio​ was associated with steatosis or fibrosis.

**Conclusions:**

Activin A was associated with liver stiffness and was higher in participants with SLD-related cirrhosis.

**Supplementary Information:**

The online version contains supplementary material available at https://doi.org/10.1186/s12876-026-05550-6.

## Background

Metabolic dysfunction-associated steatotic liver disease (MASLD) is the most common chronic liver disease, with a global prevalence nearing 40% [1]. MASLD is a multisystemic condition closely linked to the metabolic syndrome [2]. As such, it has been strongly associated with obesity and type 2 diabetes (T2D), and is defined by hepatocellular steatosis, i.e. hepatic triglyceride content exceeding 5% of total liver mass, in conjunction with one or more cardiometabolic risk factors [3]. However, nearly 20% of patients diagnosed with MASLD actually have alcohol-related liver disease (ALD) or metabolic and alcohol-related steatotic liver disease (MetALD) - etiologies associated with increased risk of developing cirrhosis and hepatocellular carcinoma (HCC), as well as increased overall mortality [4]. Although alcohol consumption is a strong disease modifier in individuals with steatotic liver disease (SLD), there seems to be a synergistic risk in the presence of cardiometabolic risk factors [5]. Though there are differences in the metabolic mechanisms driving the progression, the evident overlap of MASLD and ALD should be considered when investigating underlying molecular processes, potential biomarkers and therapeutic targets.

Previously, liver biopsy has been the gold standard for assessing liver fibrosis [6]. Non-invasive alternatives, such as transient elastography, have been developed and are widely used due to safety and patient preference. However, these methods offer improved comfort but may be less accurate for patients with obesity, significant hepatic inflammation, or ascites, which can affect measurement reliability [7]. Conversely, MR-based methods, although more precise, are associated with high operational costs and require specialized personnel, limiting their accessibility in primary healthcare settings. These limitations highlight the importance of advancing our understanding of the molecular and cellular mechanisms underlying disease progression to inform the development of future assessment strategies.

Activin A, a member of the transforming-growth factor beta (TGF-β) superfamily, has been implicated as a key mediator in hepatic fibrogenesis [8–10] (Supplementary Fig. 1). However, findings on activin A and its endogenous inhibitor follistatin in SLD are inconsistent. While some studies report increased levels of activin A [11, 12] and follistatin [11, 13] in MASLD compared to controls, others observe no significant differences in either activin A [8, 14] or follistatin [8, 12, 14]. Elevated activin A has been observed in severe alcoholic hepatitis and higher circulating activin A concentrations have been reported in alcohol-related cirrhosis compared with other cirrhosis etiologies, although MASLD-related cirrhosis has not been evaluated [15, 16]. However, most of these studies are from smaller cohorts and all are based on patients from secondary care.

We have previously demonstrated that activin A performs comparably to FIB-4 in distinguishing advanced fibrosis within a small, secondary-care cohort [17]. To build on these findings, this study further explored the levels of activin A and follistatin, in two large and more diverse populations: a primary-care cohort of patients with T2D with and without SLD, and a secondary-care cohort of patients with SLD-derived cirrhosis. The aim of this study was to explore the relationship between these two proteins and disease severity.

## Materials and methods

### Cohort characteristics

This study included participants from two Swedish multi-center studies with prospective longitudinal cohort designs: “Evaluating the prevalence and severity of NAFLD in primary care” (EPSONIP) and “A rapid, non-invasive, clinical surveillance for cachexia, sarcopenia, portal hypertension, and hepatocellular carcinoma in end-stage liver disease” (ACCESS-ESLD), previously described in detail [18–20]. The EPSONIP study included patients with T2D recruited during annual check-ups at primary health care centers in Östergötland county with the primary objective to better characterize the relationship between T2D, MASLD, hepatic fibrosis and myocardial dysfunction by using non-invasive methods based on clinical, biochemical and lifestyle profiling. To be eligible for inclusion, patients had to have a T2D diagnosis according to current guidelines and be between 35 and 75 years of age. Exclusion criteria were contraindications to perform MRI, alcohol dependence, and a previous diagnosis of hepatic cirrhosis or primary liver disease apart from MASLD.

**Fig. 1:**
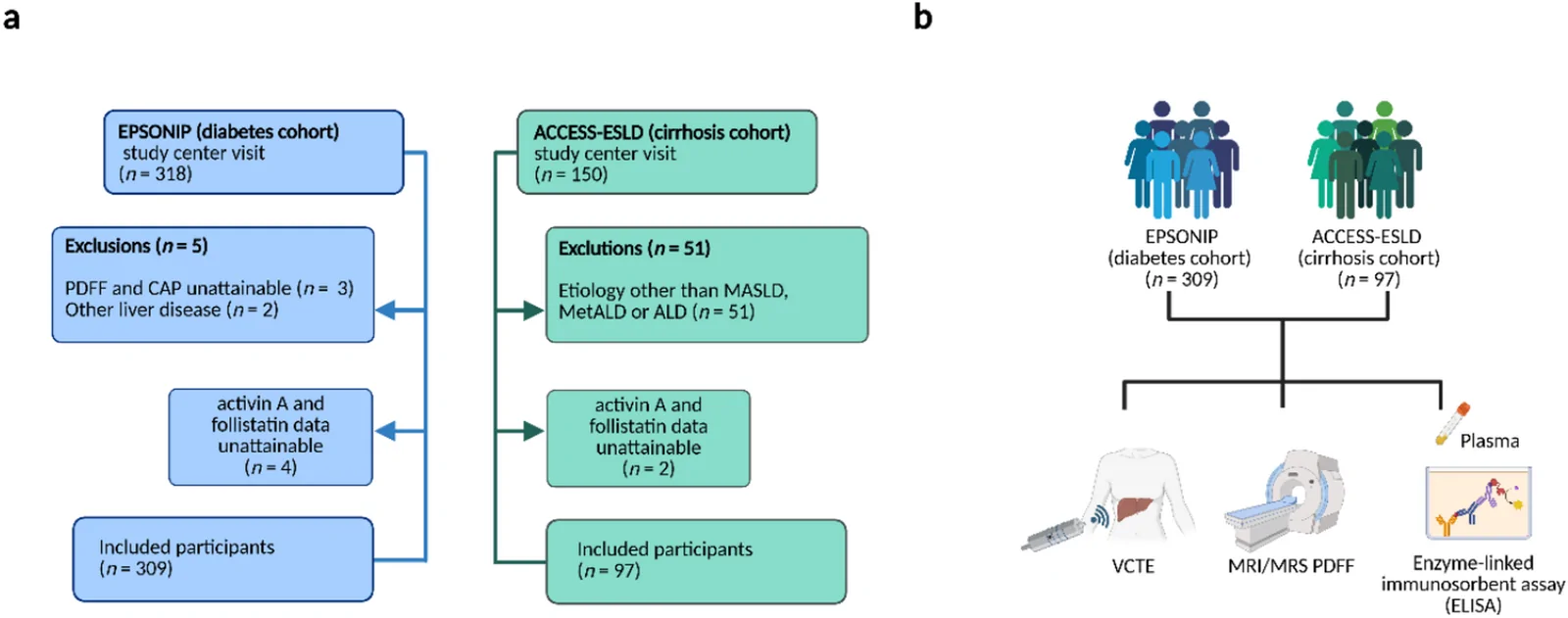
Flowchart illustrating participant enrollment (**a**) and study design (**b**). Following pre-visit exclusions, 318 EPSONIP participants attended the study center; 5 were subsequently excluded (3 for missing MASLD assessments, and 2 due to other liver diseases). Four additional exclusions were due to unavailable activin A and follistatin data, yielding 309 participants. From ACCESS-ESLD, 51 were excluded for non-MASLD/MetALD/ALD etiologies and 2 for missing biomarker data, resulting in 97 participants. In this study, EPSONIP and ACCESS-ESLD are referred to as the “diabetes cohort” and the “cirrhosis cohort”. Activin A and follistatin concentrations were analyzed with ELISA. Hepatic fat was assessed using 1 H-MRS-PDFF, MRI-PDFF, or CAP, and liver stiffness via VCTE. Abbreviations: EPSONIP: Evaluating the prevalence and severity of NAFLD in primary care, ACCESS-ESLD: A rapid, non-invasive, clinical surveillance for cachexia, sarcopenia, portal hypertension, and hepatocellular carcinoma in end-stage liver disease, MASLD: metabolic dysfunction-associated steatotic liver disease, MetALD: metabolic dysfunction–associated alcohol-related liver disease, ALD: alcohol-associated liver disease, ELISA: enzyme-linked immunosorbent assay, 1 H-MRS-PDFF: Proton Magnetic Resonance Spectroscopy–Proton Density Fat Fraction, CAP: Controlled attenuation parameter, VCTE: Vibration-controlled transient elastography

The ACCESS-ESLD study included patients with liver cirrhosis recruited at specialist health care centers for gastroenterology and hepatology in Linköping, Eksjö and Jönköping. The primary aim of the ACCESS-ESLD study was to identify risk factors and biomarkers associated with disease severity and evaluate future risk for decompensating events and hepatocellular carcinoma. All patients with established liver cirrhosis diagnosed at the age of 18 or above were eligible for inclusion. Exclusion criteria were contraindications to perform MRI, an established diagnosis of primary sclerosing cholangitis (PSC), previous HCC or liver transplantation. In the present study, we included all EPSONIP participants with available activin A measurements (*n* = 309), representing the etiologies non-MASLD, MASLD, MetALD and ALD (Fig. 1a). Additionally, all participants with SLD from the ACCESS-ESLD cohort were incorporated (*n* = 97), comprising MASLD, MetALD and ALD. The study protocols employed by EPSONIP and ACCESS-ESLD have similar baseline evaluations including among other PDFF, VCTE, bloodwork and, present and past medical history. Information regarding cirrhosis, decompensation, metabolic syndrome, hypertension, hypertriglyceridemia, and corresponding pharmacological treatments was obtained from documented medical history and current medication use at study enrolment. These variables were classified according to the following definitions. Decompensation was defined as a history of one or more of the following events: ascites, variceal bleed, jaundice, hepatic encephalopathy, or hepatorenal syndrome. Metabolic syndrome was defined in accordance with the National Cholesterol Education Program Adult Treatment Panel III criteria [21] as the presence of at least three of the following components: (1) waist circumference ≥ 40 inches in men or ≥ 35 inches in women; (2) fasting triglycerides ≥ 150 mg/dL or treatment for hypertriglyceridemia; (3) HDL cholesterol < 40 mg/dL in men or < 50 mg/dL in women or treatment for low HDL cholesterol; (4) blood pressure ≥ 130/85 mmHg or antihypertensive treatment; and (5) fasting plasma glucose ≥ 100 mg/dL or previously diagnosed T2D. Hypertension was defined as blood pressure ≥ 130/85 mmHg, measured after the participant had been resting for at least 5 min, or current antihypertensive treatment. Hypertriglyceridemia was defined as fasting triglyceride concentrations ≥ 150 mg/dL. Only cross-sectional baseline data from both studies were used in the present study. The EPSONIP and ACCESS-ESLD studies will hereafter be referred to as the “diabetes cohort” respectively the “cirrhosis cohort”.

### Blood sampling

Blood was collected in EDTA tubes from participants after an overnight fast. Within one hour of collection (while kept on ice), the blood samples were centrifuged at 2500 x g for 7 min at 4 °C. Plasma was aliquoted and stored at -80 °C until analysis.

### Immunoassay

Plasma activin A and follistatin concentrations were measured in duplicates with quantitative sandwich enzyme-linked immunosorbent assay (Fig. 1b) (R&D Systems, Minneapolis, MN, USA, DAC00B, control set QC33 and DFN00, control set QC20) in accordance with manufacturer’s instructions. Assay precision and quality control were assessed using duplicate measurements, an internal control included in each assay run, and manufacturer-provided quality control samples (QCs). Intra- and inter-assay precision were assessed using coefficients of variation (CVs), with a predefined acceptance criterion of ≤ 15%.

### Liver health measurements

Hepatic fat content was measured with proton magnetic resonance spectroscopy proton density fat fraction (^1^H-MRS-PDFF), in accordance with existing protocols [22–24], or magnetic resonance imaging-proton density fat fraction (MRI-PDFF) by AMRA Researcher (AMRA Medical AB, Linköping, Sweden). Hepatic steatosis was defined as lipid accumulation constituting a fat fraction of ≥ 5% quantified from water and fat resonances, either from a voxel of interest or from the entire liver based on a voxel-wise, quantitative estimate of liver fat content. For patients where PDFF data were not available (*n* = 29), controlled attenuation parameter (CAP) (FibroScan, Echosens, Paris, France) was used, and a value of ≥ 248 dB/m defined steatosis in accordance with current guidelines [3].

Liver stiffness measurement (LSM) was assessed with vibration-controlled transient elastography (VCTE) (FibroScan, Echosens, Paris, France), as previously described [20]. Participants fasted for ≥ 3 h before examination and measurements were considered reliable when at least 10 valid readings were obtained, with a success rate of ≥ 80% and an interquartile range ≤ 30% of the median. Examinations were initiated using the M probe and switched to the XL probe when recommended by the automated probe selection tool. Advanced fibrosis was defined as VCTE ≥ 10 kPa. The VCTE cutoffs ≥ 15 and ≥ 25 kPa were used to reflect assumed compensated advanced chronic liver disease (cACLD) and clinically significant portal hypertension (CSPH), respectively, as suggested by the renewed Baveno VII consensus [25].

Alcohol consumption (g/week) reflected self-reported current alcohol intake at study enrolment. In the diabetes cohort, MetALD was defined as having an alcohol consumption between 140 and 350 g/week (women) and 210–420 g/week (men) based on AUDIT results [3], or a phosphatidylethanol (PEth) value between 0.1 and 0.3 µmol/L, if criteria for MASLD were also fulfilled. This PEth interval falls within the range previously suggested as indicative of excessive alcohol consumption [26], while minimizing the risk of misclassifying individuals with low or sporadic alcohol consumption. ALD was defined as having an alcohol consumption above 350 g/week (women) and 420 g/week (men) and/or a PEth value above 0.3 µmol/L, regardless of other clinical or biochemical findings. In the cirrhosis cohort, liver disease etiology was determined according to the original clinical assessment and documented medical history at study enrolment. As many participants had reduced or ceased alcohol consumption following medical advice, current alcohol intake at study enrolment may not reflect the historical alcohol exposure underlying disease development.

### Statistical analysis

Data is represented as median and interquartile range (IQR) or total numbers and percentages when applicable. All statistical analysis was performed using SPSS IBM Statistics, version 29.0 (IBM Corp, Armonk, NY, USA), with listwise deletion applied to exclude cases with missing values on any of the analyzed variables, and GraphPad Prism version 10.0.2 (GraphPad Software, Boston, MA, USA). Figures were made with BioRender. Since most continuous variables were not normally distributed non-parametric statistical tests were used for group comparisons and correlation analyses; the Mann Whitney *U* and Kruskal Wallis tests were used for continuous variables, and the Chi^2^ test was used for nominal data. Spearman’s correlation coefficient (ρ) was used to assess correlation. Rank-transformed ANCOVA was used to adjust for fibrosis when comparing participants from the diabetes cohort with steatosis to those without. In addition, multivariable linear regression was performed to assess whether the association between activin A concentrations and liver stiffness remained after adjustment for potential confounding factors. Probability values were considered significant at *P* < 0.05.

## Results

### Cohort characteristics

**Fig. 2:**
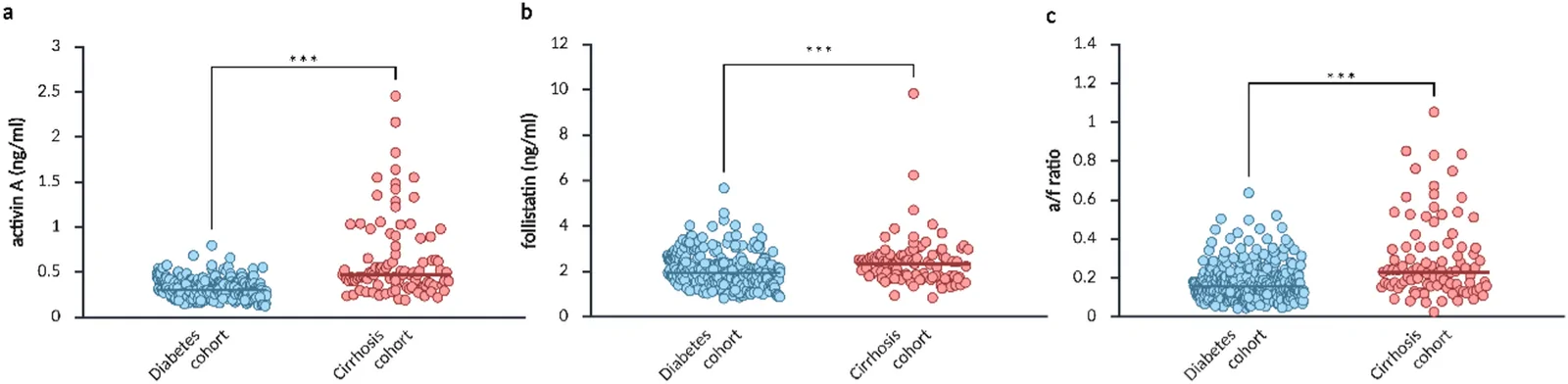
Plasma concentrations of activin A (**a**), follistatin (**b**), activin A-to-follistatin (a/f) ratio (**c**), in participants from the diabetes cohort compared to the cirrhosis cohort. Data are presented as individual values and median. Mann–Whitney U test was used for pairwise comparisons between groups in panels a-c. Probability values were considered significant at *P* < 0.05. Asterisks denote statistical significance: *** = *P* < 0.001. Abbreviations: a/f: activin/follistatin ratio

The diabetes and cirrhosis cohort, comprising 309 and 97 individuals respectively, displayed comparable age (66.0 vs. 64.0 years, *P* = 0.981), BMI (28.7 vs. 29.0, *P* = 0.873), and sex distribution (37% vs. 39% females, *P* = 0.194) (Table 1). The levels of activin A (0.30 ng/mL vs. 0.49 ng/mL, *P* < 0.001), follistatin (1.90 ng/mL vs. 2.33 ng/mL, *P* < 0.001) and their ratio (a/f) (0.162 vs. 0.239, *P* < 0.001) were significantly higher in the cirrhosis cohort compared to the diabetes cohort (Fig. 2a-c).

**Table 1:** Cohort characteristics

	EPSONIP	ACCESS-ESLD	
	*n* = 309	*n* = 97	*P*
**Demographic and clinical**			
Sex (female)	113 (37%)	38 (39%)	0.194
Age (years) (*n* = 299/95)	66.0 (10.0)	64.0 (11.5)	0.981
BMI (kg/m^2^) (*n* = 308/97)	28.7 (6.18)	29.0 (6.55)	0.873
Overweight (≥ 25 kg/m^2^) (*n* = 308/97)	256 (83%)	78 (80%)	0.584
Obese (≥ 30 kg/m^2^) (*n* = 308/97)	138 (45%)	44 (45%)	0.904
Time since diagnosis (years) (*n* = 308/97)	11.0 (9.75)	5.00 (4.00)	**< 0.001**
Waist circumference (cm) (*n* = 300/96)	104 (16.8)	104 (13.5)	0.187
Waist to hip ratio (*n* = 297/96)	0.985 (0.0939)	0.991 (0.0824)	0.672
Alcohol (g/week) (*n* = 309/92)	13.5 (40.5)	0.00 (24.0)	**< 0.001**
Hypertension	204 (66%)	62 (64%)	0.704
Systolic BP (mm Hg) (*n* = 303/97)	130 (18.0)	140 (23.0)	**< 0.001**
Diastolic BP (mm Hg) (*n* = 302/97)	78.0 (15.0)	85.0 (11.0)	**< 0.001**
Carvedilol treatment	1 (0.32%)	27 (28%)	-
Dyslipidemia (*n* = 309/96)	233 (75%)	38 (39%)	**< 0.001**
Statin treatment (*n* = 288/97)	231 (80%)	39 (40%)	**< 0.001**
Metabolic syndrome	309 (100%)	48 (50%)	**< 0.001**
Glucose lowering therapy	284 (92%)	46 (47%)	**< 0.001**
Lifestyle modification	97 (31%)	19 (20%)	**0.025**
Oral medication	260 (84%)	40 (41%)	**< 0.001**
Insulin treatment (*n* = 299/97)	64 (21%)	22 (23%)	0.791
Combination therapy	284 (92%)	29 (30%)	**< 0.001**
Decompensation	N/A	37 (38%)	-
Child Pugh score 5 (*n* = 0/95)	N/A	73 (77%)	-
Child Pugh score 6 (*n* = 0/95)	N/A	13 (14%)	-
Child Pugh score 7 (*n* = 0/95)	N/A	7 (7%)	-
Child Pugh score 8 (*n* = 0/95)	N/A	1 (1%)	-
Child Pugh score 9 (*n* = 0/95)	N/A	1 (1%)	-
**Biochemical**			
FIB-4	1.36 (0.717)	3.21 (2.49)	**< 0.001**
Prothrombin (INR) (*n* = 301/97)	1.00 (0.100)	1.10 (0.20)	**< 0.001**
Platelet count (10^9^/L) (*n* = 307/97)	225 (86.3)	142 (71.5)	**< 0.001**
AST (µkat/L) (*n* = 308/95)	0.400 (0.148)	0.660 (0.400)	**< 0.001**
ALT (µkat/L)	0.430 (0.220)	0.610 (0.430)	**< 0.001**
ALP (µkat/L)	1.10 (0.350)	1.70 (0.90)	**< 0.001**
ƔGT (µkat/L)	0.420 (0.330)	2.40 (3.60)	**< 0.001**
Bilirubin (µmol/L) (*n* = 308/97)	8.00 (6.00)	13.0 (9.50)	**< 0.001**
Albumin (g/L)	42.0 (4.00)	39.0 (6.00)	**< 0.001**
Glucose (mmol/L) (*n* = 301/95)	7.50 (1.90)	6.20 (1.50)	**< 0.001**
Creatinine (µmol/L) (*n* = 308/97)	72.0 (21.0)	71.0 (21.0)	0.440
Cholesterol (mmol/L) (*n* = 309/95)	3.80 (1.20)	4.00 (1.75)	0.210
Triglycerides (mmol/L) (*n* = 309/95)	1.20 (0.768)	1.30 (0.815)	0.910
LDL (mmol/L) (*n* = 301/94)	1.80 (0.975)	2.20 (1.35)	**0.007**
HDL (mmol/L) (*n* = 309/95)	1.30 (0.400)	1.30 (0.550)	0.396
Insulin (mIE/L) (*n* = 306/97)	14.0 (10.2)	23.0 (22.0)	**< 0.001**
Activin A (ng/mL)	0.30 (0.119)	0.49 (0.48)	**< 0.001**
Follistatin (ng/mL) (*n* = 304/97)	1.90 (0.807)	2.33 (1.03)	**< 0.001**
Activin to Follistatin ratio	0.162 (0.104)	0.239 (0.198)	**< 0.001**
**Imaging**			
PDFF (%) (*n* = 280/97)	5.36 (10.3)	4.03 (6.08)	0.075
Steatosis	181 (59%)	40 (41%)	**0.003**
CAP (dB/m) (*n* = 297/62)	282 (92)	273 (68)	0.294
VCTE (kPa) (*n* = 305/82)	5.10 (2.30)	21.6 (26.9)	**< 0.001**
VCTE ≥ 8 kPa	44 (14%)	78 (95%)	**< 0.001**
VCTE ≥ 10 kPa	21 (7%)	74 (90%)	**< 0.001**
VCTE ≥ 15 kPa	5 (1.6%)	58 (70%)	**< 0.001**
VCTE ≥ 25 kPa	3 (1%)	38 (46%)	**< 0.001**

Demographic, clinical and biochemical characteristics of participants, divided by cohort. Continuous values are presented as median (IQR) or n (%) when applicable. Probability values were considered significant at *P* < 0.05. Categorical variables were tested with Chi^2^-test and continuous variables with Mann–Whitney U test. Bold values indicate statistically significant differences. Values in parentheses indicate the number of observations (EPSONIP/ACCESS-ESLD) when different from the total cohort size

Abbreviations: BMI: Body mass index, BP: Blood pressure, CVD: Cardiovascular disease, FIB-4: Fibrosis-4 index, Prothrombin INR: International normalized ratio, AST: Aspartate aminotransferase, ALT: Alanine aminotransferase, ALP: Alkaline phosphatase, ɣGT: Gamma-glutamyltransferase, LDL: Low-density lipoprotein, HDL: High-density lipoprotein, PDFF: Proton density fat fraction, CAP: Controlled attenuation parameter, VCTE: Vibration-controlled transient elastography

### Hepatic steatosis does not affect activin A or follistatin plasma concentrations

**Fig. 3:**
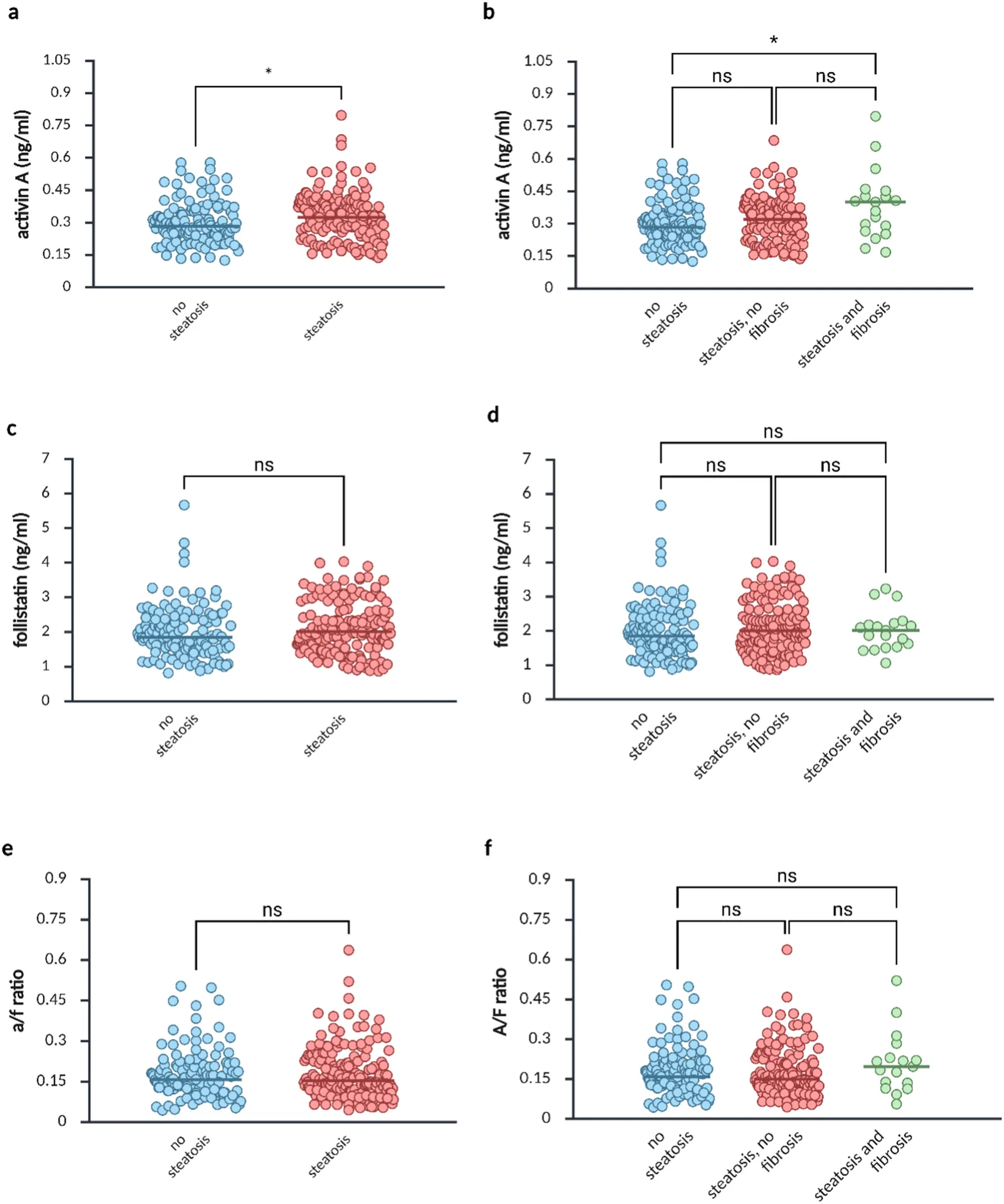
Plasma concentrations of activin A (**a**-**b**), follistatin (**c**-**d**), activin A-to-follistatin (a/f) ratio (**e-f**), in participants from the diabetes cohort, stratified by the presence or absence of steatosis and fibrosis. Steatosis was defined as PDFF ≥ 5% or, if unavailable, CAP ≥ 248 dB/m. Fibrosis was defined as VCTE ≥ 10 kPa. Data are presented as individual values and median. Mann–Whitney U test was used for pairwise comparisons between groups (panels a, c and e) and an omnibus test was performed using Kruskal–Wallis (panels b, d, f), followed by post-hoc pairwise comparisons using Dunn’s test. Probability values were considered significant at *P* < 0.05. Asterisks denote statistical significance: * = *P* < 0.05. Abbreviations: a/f: activin/follistatin ratio, PDFF: Proton density fat fraction, CAP: Controlled attenuation parameter, VCTE: Vibration-controlled transient elastography

To investigate the role of activin A and follistatin in steatosis, we stratified the diabetes cohort based on hepatic fat content, in which 59% of participants had liver steatosis. Fasting plasma activin A concentrations were higher in participants with steatosis compared to those without (0.325 ng/mL vs. 0.285 ng/mL, *P* < 0.05) (Fig. 3a). The significance was, however, lost when adjusting for LSM obtained from VCTE. To further investigate the association between fibrosis and activin A levels, we stratified participants in the diabetes cohort by the presence or absence of advanced fibrosis. Activin A concentrations differed across the three groups (Kruskal-Wallis, *P* = 0.023). Post-hoc analysis demonstrated higher activin A concentrations in participants with steatosis and fibrosis compared with those without both steatosis and fibrosis (0.400 vs. 0.285 ng/mL, *P* = 0.036), whereas differences between the steatosis groups with and without fibrosis did not reach statistical significance (Fig. 3b). Follistatin concentrations did not differ significantly between participants with and without steatosis (2.01 ng/mL vs. 1.85 ng/mL, *P* = 0.069) (Fig. 3c) and remained unchanged in the presence of advanced fibrosis (2.02 ng/mL vs. 2.00 ng/mL vs. 1.85 ng/mL, *P* = 0.244) (Fig. 3d). A similar pattern was observed for the activin A-to-follistatin ratio (0.153 vs. 0.158, *P* = 0.856 and 0.197 vs. 0.149 vs. 0.158, *P* = 0.529) (Figs. 3e–f).

We next explored the association between hepatic fat content and activin A concentrations in the cirrhosis cohort. Activin A concentrations were significantly lower in participants with steatosis compared to those without (0.439 ng/mL vs. 0.513 ng/mL, *P* = 0.022) (Supplementary Fig. 2a). Although correlation analysis revealed an inverse relationship between activin A and PDFF, this association was weak (ρ = -0.277, *P* = 0.006) (Supplementary Fig. 2b).

### Plasma activin A concentrations are associated with markers of advanced liver disease

**Fig. 4:**
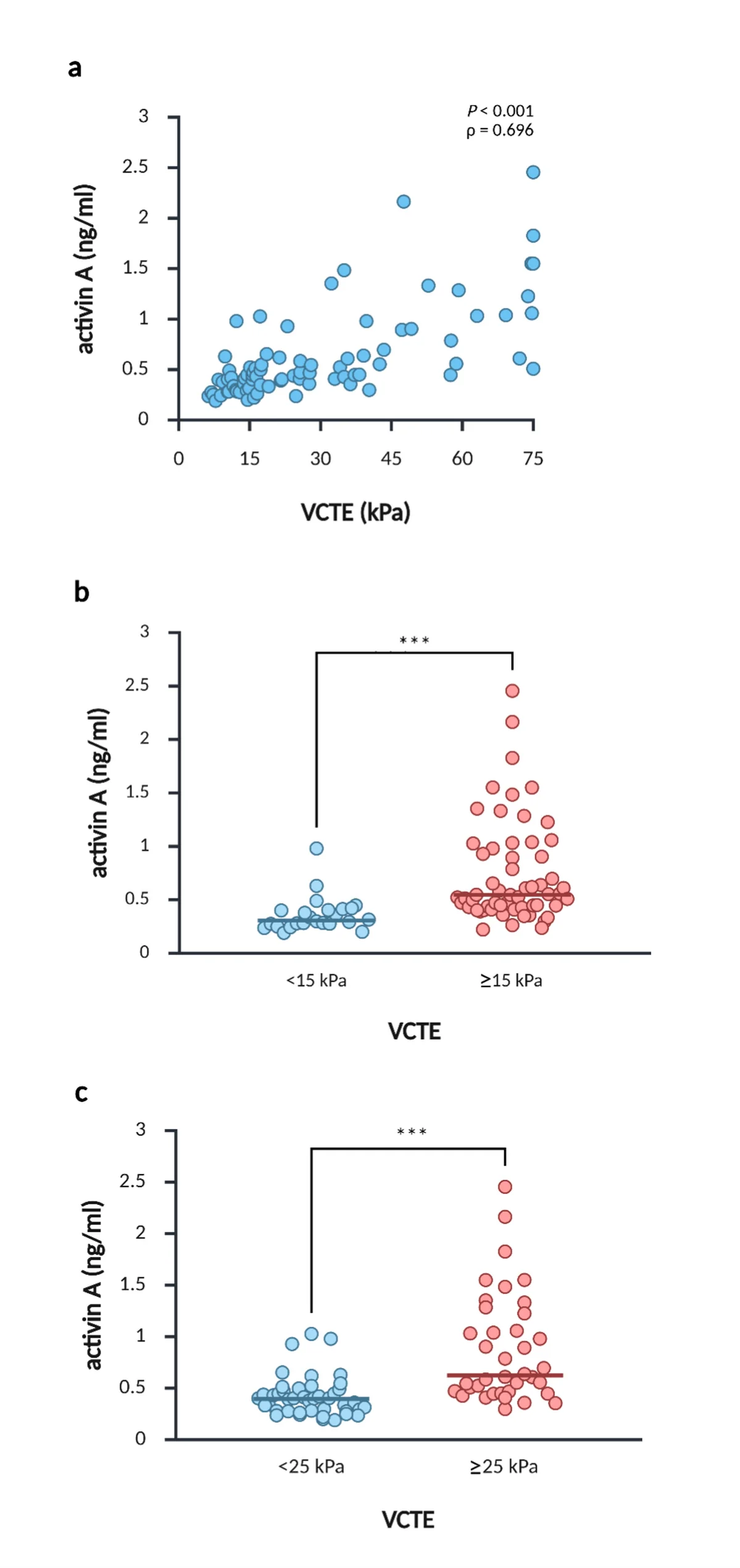
Correlation between VCTE (kPa) values and activin A (**a**) and plasma concentrations of activin A in participants stratified by VCTE suggestive of cACLD and CSPH in the cirrhosis cohort (**b**-**c**). Data are presented as individual values and median. Spearman’s Rho was used for bivariate correlation analysis in panel a, Mann–Whitney U test for pairwise comparisons between groups in panels b-c. Probability values were considered significant at *P* < 0.05. Asterisks denote statistical significance: *** = *P* < 0.001. Abbreviations: VCTE: Vibration-controlled transient elastography, cACLD: compensated advanced chronic liver disease, CSPH: Clinically significant portal hypertension

To further investigate the relationship between activin A and fibrosis, we assessed its correlation with liver stiffness. In the diabetes cohort, the correlation between activin A and VCTE (*n* = 305) was weak (ρ = 0.134, *P* = 0.019). However, when the same analysis was performed in the cirrhosis cohort in participants with available VCTE data (*n* = 82), we observed a strong positive correlation between activin A and liver stiffness (ρ = 0.696, *P* < 0.001) (Fig. 4a). In a multivariable linear regression model including age, sex, BMI, serum creatinine, and ALD etiology, liver stiffness remained independently associated with activin A concentrations (B = 0.015, 95% CI: 0.011–0.019, *P* < 0.001). Moreover, activin A concentrations were significantly higher in participants with VCTE suggestive of cACLD than in those without (0.546 ng/mL vs. 0.307 ng/mL, *P* < 0.001) (Fig. 4b) and were similarly elevated in participants with VCTE suggestive of CSPH (0.624 ng/mL vs. 0.396 ng/mL, *P* < 0.001) (Fig. 4c).

### Ability of activin A to classify fibrosis in early MASLD

To investigate activin A’s potential role in classifying fibrosis in early MASLD pathology, we evaluated its discriminatory capacity in the diabetes cohort alongside the commonly used FIB-4 score. The ability of activin A to classify participants with VCTE ≥ 8 kPa and ≥ 10 kPa was modest (Area under the receiver operating characteristic curve (AUROC) = 0.640, 95% CI: 0.553–0.727 and 0.653, 95% CI: 0.5180.788, respectively) with AUROC estimates similar to those observed for FIB-4 (AUROC = 0.611, 95% CI: 0.518–0.703 and 0.637, 95% CI: 0.513–0.760, respectively) (Supplementary Fig. 3a-b). Among participants with obesity (*n* = 138), activin A showed numerically higher AUROC estimates for both ≥ 8 kPa and ≥ 10 kPa (AUROC = 0.656, 95% CI: 0.541–0.770 and 0.702, 95% CI: 0.545–0.857), whereas the corresponding AUROC estimates for FIB-4 were 0.641 (95% CI: 0.531–0.751) and 0.636 (95% CI: 0.498–0.774), respectively.

### Activin A in ALD

To investigate whether plasma activin A concentrations differed according to liver disease etiology, we compared plasma activin A concentrations between participants with MASLD and ALD in the cirrhosis cohort. Because the number of participants with cirrhosis derived from MetALD was too few, these were not included in the statistical analysis. Although activin A was elevated in participants with cirrhosis derived from ALD compared to MASLD (Supplementary Fig. 4), this result did not reach statistical significance (0.693 ng/mL vs. 0.556 ng/mL, *P* = 0.180). We then proceeded by investigating whether activin A correlated with the alcohol biomarker phosphatidylethanol (PEth). This analysis did not reach statistical significance in either the diabetes cohort (ρ = 0.032, *P =* 0.573) or in the cirrhosis cohort (ρ = 0.076, *P =* 0.524).

## Discussion

In this study, we demonstrate that activin A concentrations correlate with LSM in individuals with advanced chronic liver disease. In addition, higher activin A concentrations were observed in participants with more advanced stages of SLD.

Activin A has previously been linked to hepatic fat content and suggested to exert anti-steatotic functions in early MASLD etiology by upregulating hepatic mitochondrial β-oxidation of fatty acids and downregulating *de novo* fatty acid synthesis [11]. Overexpression of activin A in MASLD/Metabolic dysfunction-associated steatohepatitis (MASH) mice models dramatically mitigated steatosis and systemic fat accumulation [27]. Moreover, elevated serum activin A has been observed in individuals with simple steatosis compared to controls [11], although most studies report unaffected activin A concentrations in simple steatosis [12, 14, 28]. In the diabetes cohort, we observed slightly higher activin A levels in individuals with SLD compared to those without. When we stratified participants with SLD by fibrosis status, the difference was evident only between those with SLD and advanced fibrosis compared to those without SLD. Activin A concentrations were higher in the cirrhosis cohort, which is in accordance with previous research [8, 11, 12, 17], although in one study the association was lost after adjusting for adiposity [12]. Moreover, the correlation of activin A with liver stiffness strengthened from weak in the diabetes cohort to strong in the cirrhosis cohort, while its correlation with PDFF shifted from a weak positive to a weak negative. Taken together, these observations may indicate that circulating activin A concentrations are more strongly associated with fibrosis-related disease characteristics than with steatosis. This interpretation is supported by previous experimental studies linking activin A signalling to hepatic fibrogenesis and extracellular matrix deposition.

A hallmark of advanced disease is typically a regression in steatosis, accompanied by a progressive increase in fibrosis. LSM are in addition to fibrosis, affected by portal hypertension. Accordingly, activin A levels were significantly higher in participants with VCTE suggestive of (CSPH) compared to those with lower liver stiffness, suggesting that activin A may also reflect the hemodynamic consequences of cirrhosis.

In hepatocytes, follistatin synthesis is induced by activin A, constituting a feedback mechanism in which activin A promotes expression of its own inhibitor [29]. In contrast, activated hepatic stellate cells produce high levels of activin A without a corresponding follistatin upregulation, suggesting cell-specific dysregulation of this feedback loop in fibrotic disease [30]. Previous reports on follistatin in MASLD have been inconsistent. While some studies report higher follistatin levels in simple steatosis and others show no difference compared with controls, circulating concentrations appear to be strongly influenced by adiposity [8, 11, 12, 14]. In contrast, most studies report increased follistatin with advancing fibrosis [11, 14]. Consistent with the majority of reports, we observed no significant differences in follistatin levels related to steatosis within the diabetes cohort. We further found higher follistatin levels in the cirrhosis cohort compared with the diabetes cohort. Although no fibrosis‑related differences were observed within the diabetes cohort, this may reflect the limited number of participants with advanced fibrosis and the confounding effect of high BMI.

Some studies have reported an increasing activin A–to–follistatin ratio with disease progression [8, 11] while others report the opposite [14]. We found the ratio to be higher in the cirrhosis cohort, whereas no significant differences were observed between participants with and without advanced fibrosis within the diabetes cohort. Together, the parallel elevation of activin A and follistatin could suggest a predominant upregulation of activin A with relatively insufficient follistatin‑mediated inhibition, supporting a dysregulated activin A–follistatin axis in advanced disease.

Activin A has previously been shown to be elevated in MASLD cirrhosis compared to non-cirrhotic conditions and to be further increased in alcohol-related cirrhosis [8, 15, 16]. Furthermore, patients with alcohol-related cirrhosis have been shown to exhibit higher circulating activin A concentrations than patients with viral or primary biliary cirrhosis [16]. Elevated activin A in alcohol-related cirrhosis is believed to be due to its more rapid progression compared to other etiologies. However, we did not observe any differences in plasma activin A when comparing participants with cirrhosis derived from MASLD and ALD. Although there was a trend towards increased activin A in participants with alcohol-related cirrhosis, this did not reach statistical significance.

The main strength of our study is our highly phenotyped cohorts, encompassing patients with a broad spectrum of SLD and fibrosis. By incorporating participants from a relatively healthy cohort, predominantly exhibiting early MASLD features, alongside individuals representing advanced stages of MASLD and ALD, we were able to examine the associations of activin A and follistatin with steatosis and fibrosis across the full spectrum of disease severity. Patients were recruited both in primary and secondary care, included in a prospective manner and diagnosed according to current guidelines. In addition, both cohorts adhered to a largely uniform study protocol [18, 19].

Nevertheless, our study has some limitations. The use of liver biopsies for fibrosis staging would have contributed valuable insight into the relationship between activin A and fibrosis. However, fibrosis severity was approximated using VCTE-derived liver stiffness measurements, which represent a surrogate marker of fibrosis rather than direct histological assessment. As liver stiffness may be influenced by factors other than fibrotic burden, the observed associations may have been attenuated. Nevertheless, VCTE remains the most widely used and best-validated non-invasive tool for fibrosis assessment. Moreover, the relatively high prevalence of obesity in the cirrhosis cohort (approximately 45%) should also be considered when interpreting the CSPH-related findings. In particular, the applicability of the Baveno VII ≥ 25 kPa threshold may be less certain in patients with obesity and MASLD than in the populations for which this threshold is recommended, including patients with viral- or alcohol-related cACLD and non-obese patients with NASH-related cACLD. Future longitudinal studies are warranted to determine whether the associations observed between activin A and liver stiffness translate into clinically relevant outcomes. Furthermore, healthy selection bias remains a persistent concern in prospective trials, even when broad inclusion criteria and minimal exclusions are applied. In the diabetes cohort, this was suggested by the relatively low BMI (28.7). Similarly, in the cirrhosis cohort, the high frequency of participants with Child-Pugh A5 (77%) could infer a selection of participants with either low risk of advanced fibrosis or low risk of decompensated cirrhosis.

## Conclusions

The findings of this study suggest that circulating activin A concentrations are more strongly associated with fibrosis-related disease characteristics and advanced stages of SLD than with steatosis, regardless of disease etiology. Although activin A participates in multiple physiological and pathological processes, its reported involvement in hepatic fibrogenesis, together with the findings of the present study, warrants further mechanistic and longitudinal investigations into the role of activin A in advanced SLD.

## Supplementary Information

Below is the link to the electronic supplementary material.

Supplementary Material 1

## Data Availability

The datasets generated and/or analyzed during the current study are not publicly available due to ethical and institutional restrictions but are available from the corresponding author upon request.

## Abbreviations

a/f: Activin A-to-Follistatin ratio
ACCESS-ESLD: A rapid, non-invasive, clinical surveillance for CachExia, sarcopenia, portal hypertension, and hepatocellular carcinoma in end-stage liver disease
ALD: Alcohol-associated liver disease
ALP: Alkaline phosphatase
ALT: Alanine aminotransferase
AST: Aspartate aminotransferase
AUD: Alcohol Use Disorder
AUDIT: Alcohol Use Disorders Identification Test
AUROC: Area Under the Receiver Operating Characteristic Curve
BMI: Body Mass Index
CAP: Controlled Attenuation Parameter
cACLD: Compensated Advanced Chronic Liver Disease
CV: Coefficient of variation
CSPH: Clinically Significant Portal Hypertension
EDTA: Ethylenediaminetetraacetic Acid
ELISA: Enzyme-Linked Immunosorbent Assay
EPSONIP: Evaluating the Prevalence and Severity of NAFLD in Primary Care
FIB-4: Fibrosis-4 Index
HCC: Hepatocellular Carcinoma
LSM: Liver Stiffness Measurement
MASLD: Metabolic Dysfunction-Associated Steatotic Liver Disease
MASH: Metabolic Dysfunction-Associated Steatohepatitis
MetALD: Metabolic Dysfunction-Associated Alcohol-Related Liver Disease
MRI: Magnetic Resonance Imaging
MRS: Magnetic Resonance Spectroscopy
NASH: Non-Alcoholic Steatohepatitis
PDFF: Proton Density Fat Fraction
PEth: Phosphatidylethanol
PSC: Primary Sclerosing Cholangitis
QCs: Quality Controls
SLD: Steatotic Liver Disease
T2D: Type 2 Diabetes
TGF-β: Transforming Growth Factor Beta
VCTE: Vibration-Controlled Transient Elastography
ɣGT: Gamma-Glutamyl Transferase
