# Supplementary material for: Elevated circulating activin A is associated with disease severity in advanced steatotic liver disease (SLD)
Source: BMC Gastroenterol. 2026 Nov 10;26:750. doi: 10.1186/s12876-026-05550-6 (PMC13749851; doi:10.1186/s12876-026-05550-6)
Supplement: Supplementary file 1 [file 12876_2026_5550_MOESM1_ESM.docx]

Supplementary

***Supplementary Figure 1. Proposed mechanisms linking activin A signalling to hepatic fibrogenesis.*** *Experimental studies suggest that, following inflammatory stimuli, hepatocytes secrete activin A into the extracellular space. Activin A is believed to promote Kupffer cell-mediated activation of HCs, which, upon adopting a myofibroblast-like phenotype, contribute to fibrosis through extracellular matrix and collagen deposition and increased activin A production. Under normal conditions, activin A induces follistatin, its endogenous inhibitor, as part of a negative feedback mechanism; however, this regulatory pathway has been suggested to be impaired in fibrotic disease.*

*Abbreviations: HCs; hepatic stellate cells*

***Supplementary figure 2. Plasma concentrations of activin A stratified by the presence or absence of steatosis (a) and correlated with PDFF values (b) in the cirrhosis cohort.*** *Steatosis was defined as PDFF ≥ 5 % or, if unavailable, CAP ≥ 248 dB/m. Data are presented as individual values and median. Mann–Whitney U test was used for pairwise comparisons between groups in panel a and Spearman’s rho was used for bivariate correlation analysis in panel b. Probability values were considered significant at P < 0.05. Asterisks denote statistical significance: * = P < 0.05.*

*Abbreviations: PDFF: Proton density fat fraction, CAP: Controlled attenuation parameter*

***Supplementary figure 3. ROC curves comparing FIB-4 and activin A for classifying fibrosis in the diabetes cohort, stratified by VCTE thresholds ≥ 8 kPa (a) and ≥ 10 kPa.*** *Sensitivity (%) is plotted against 100 − specificity (%). The diagonal line represents the line of no discrimination (AUROC = 0.5).*

*Abbreviations: ROC: Receiver operating curve, FIB-4: Fibrosis-4 index, VCTE: Vibration-controlled transient elastography, AUROC: Area under the receiver operating characteristic curve*

 ***Supplementary figure 4. Plasma concentrations of activin A in participants with MASLD or ALD in the cirrhosis cohort.*** *Data is presented as individual values and median. Probability values were considered significant at P < 0.05. Pairwise comparisons and statistical association were assessed with Mann–Whitney U test.*

*Abbreviations: MASLD: metabolic-dysfunction associated steatotic liver disease, ALD: alcohol-associated liver disease*
